# Lectin histochemistry of *Kudoa septempunctata* genotype ST3-infected muscle of olive flounder (*Paralichthys olivaceus*)

**DOI:** 10.1051/parasite/2016021

**Published:** 2016-05-11

**Authors:** Jaeyoun Kang, Changnam Park, Yeounghwan Jang, Meejung Ahn, Taekyun Shin

**Affiliations:** 1 College of Veterinary Medicine, Jeju National University Jeju 63243 Republic of Korea; 2 Incheon International Airport Regional Office, National Fishery Products Quality Management Service, Ministry of Oceans and Fisheries Incheon 22382 Republic of Korea; 3 Ocean and Fisheries Research Institute, Jeju Special Self-Governing Province, Pyoseon-myeon Segwipo-si, Jeju 63629 Republic of Korea; 4 College of Medicine, Jeju National University Jeju 63243 Republic of Korea

**Keywords:** *Kudoa septempunctata*, Lectin, Myxozoa, Olive flounder

## Abstract

The localization of carbohydrate terminals in *Kudoa septempunctata* ST3-infected muscle of olive flounder (*Paralichthys olivaceus*) was investigated using lectin histochemistry to determine the types of carbohydrate sugar residues expressed in *Kudoa* spores. Twenty-one lectins were examined, i.e., N-acetylglucosamine (s-WGA, WGA, DSL-II, DSL, LEL, STL), mannose (Con A, LCA, PSA), galactose/N-acetylgalactosamine (RCA12, BSL-I, VVA, DBA, SBA, SJA, Jacalin, PNA, ECL), complex type N-glycans (PHA-E and PHA-L), and fucose (UEA-I). Spores encased by a plasmodial membrane were labeled for the majority of these lectins, with the exception of LCA, PSA, PNA, and PHA-L. Four lectins (RCA 120, BSL-I, DBA, and SJA) belonging to the galactose/N-acetylgalactosamine group, only labeled spores, but not the plasmodial membrane. This is the first confirmation that various sugar residues are present in spores and plasmodial membranes of *K. septempunctata* ST3.

## Abstract (fr)

La localisation des terminaux de carbohydrates dans le muscle du cardeau hirame (*Paralichthys olivaceus*) infecté par *Kudoa septempunctata* génotype ST3 a été étudiée en utilisant l’histochimie des lectines, afin de déterminer les types de résidus d’hydrate de carbone exprimés dans les spores de *Kudoa*. Vingt-et-une lectines ont été examinées, à savoir N-acétylglucosamine (s-WGA, WGA, DSL-II, DSL, LEL, STL), mannose (Con A, LCA, PSA), galactose/N-acétylgalactosamine (RCA12, BSL-I, VVA, DBA, SBA, SJA, jacaline, PNA, ECL), glycanes complexes de type N (PHA-E et PHA-l) et fucose (UEA-I). Les spores enveloppées dans une membrane plasmodiale ont été marquées par la majorité de ces lectines, à l’exception de LCA, PSA, PNA et PHA-l. Quatre lectines (RCA 120, BSL-I, DBA et SJA), appartenant au groupe galactose/N-acétylgalactosamine, ont marqué seulement les spores mais pas la membrane plasmodiale. Ceci est la première confirmation que divers résidus de sucre sont présents dans les spores et les membranes plasmodiales de *K. septempunctata* ST3.

## Introduction

*Kudoa septempunctata* Matsukane et al., 2010 [9], a myxosporean species of the order Multivalvulida, has been identified in the trunk muscle of aquacultured olive flounder (*Paralichthys olivaceus*), occasionally causing food poisoning in Japan [8, 9]. *Kudoa septempunctata*-infected raw olive flounder fillets have occasionally reached diners’ tables because the infection was not grossly identified. Even though the life cycle of *K. septempunctata* has not been clarified within or outside of olive flounders, it has been reported that *Kudoa* species is maintained between oligochaete and fish [18]. After infection of fish by *Kudoa* species, it is suggested that they move to the tissues of preference and develop into a plasmodium [1, 3].

*Kudoa septempunctata* spores are composed of six or seven shell valves and polar capsules [9], which are genetically classified into three groups, i.e. ST1, ST2, and ST3. Both ST1 and ST2 are common in Japan, while ST3 is dominant in the Republic of Korea [17]. Despite the distinct genetic differences among *K. septempunctata*, infected tissues show similar parasite presentation in that spores develop within pseudocysts in the muscle fibers of infected flat fish [11]. However, no inflammatory lesions are found either in or around infected muscle fibers.

Carbohydrate residues detected by lectin histochemistry are widely localized on epithelial cells in flat fish, where they play important roles in protecting the organism [6]. It was recently reported that carbohydrate residues are involved in protecting the organism against the environment and protecting parasites from the host as decoys for host immune cells [15]. Thus, carbohydrate residues are important factors for the interaction between host cells and parasites participating in the adhesion and penetration of parasites [13]. Carbohydrate residues on the spores of myxozoan parasites (*Myxobolus cerebralis*) have been characterized through lectin histochemistry [7]. A previous study using a limited number of lectins (WGA, SBA, BS-I, Con A, UEA-I, and SNA) in several myxosporean parasite infections showed that lectin reactivity revealed different binding patterns [10], which would be useful in diagnostic studies [10].

The present study was performed to evaluate the characteristics of carbohydrate residues on spores of *K. septempunctata* genotype ST3 in infected muscle of cultured olive flounder, an abundant genotype in Korea.

## Materials and methods

### Sample collection

Olive flounder (*Paralichthys olivaceus*) is one of the most important aquaculture fish in Korea. Olive flounder has been cultured in 10 ton flow-through land-tanks at 22 °C ± 1 under a natural photoperiod. Supplemental aerations were provided to maintain dissolved oxygen levels near 7.0 ± 0.5 ppm and the salinity was 32 ± 1 ppt. *Kudoa septempunctata*-infected fish were periodically screened by microscopic examination of crude suspensions of muscle tissue at 400× magnification. To screen for parasite infection, fish were anesthetized in buffered 3-aminobenzoic acid ethyl ester methanesulfonate (Sigma). *Kudoa septempunctata* infection in flat fish was further confirmed by histological examination, as reported previously [2].

### Histological studies

Muscle samples of *K. septempunctata*-infected fish were fixed in 10% neutral buffered formalin and processed for embedding in paraffin. The paraffin-embedded tissues were cut into sections at a thickness of 5 μm using a rotary microtome (Leica, Nussloch, Germany). The tissue sections were stained with hematoxylin and eosin for routine histopathological examination. The histological findings were reported in our previous report [2], and some of the samples diagnosed by PCR were also used for lectin histochemistry in the present study.

### Detection of *Kudoa* spores in muscles

DNA was extracted from the infected muscle in flounder fish using a QIAamp DNA Mini Kit (Qiagen, Venlo, Netherlands), following the manufacturer’s instructions. Conventional PCR primers were designed to detect two *K. septempunctata* mitochondrial genes: cytochrome *c* oxidase subunit I (*cox1*; 751 bp) and large subunit rRNA (*rnl*; 817 bp) [17]. Briefly, the primers used were *cox 1*-F1 (5′-TTTGTTCATCGGCACAATTC-3′), *cox 1*-R1 (5′-ATAGCCTGGAACAAGGAATC-3′), *rnl*-F1 (5′-TGCCGTCATTCTGTTGTATT-3′), and *rnl*-R1 (5′-AATACCCATGCTGTGTTCAT-3′) as described in a previous paper [2, 17]. PCR amplification was performed in a 25 μL reaction volume containing 2 μL genomic DNA, 15 pmol of each primer, and Diastar^TM^ Taq DNA polymerase (Solgent, Korea), following the manufacturer’s protocol. PCR amplifications were performed with initial denaturation (95 °C, 3 min) followed by 30 cycles of denaturing (95 °C, 30 s), annealing (55 °C, 30 s), and extension (60 °C, 60 s), with a final extension (68 °C, 5 min). Both negative control without template DNA and positive control (plasmid) [2] were included to check for contamination and primer specificity, respectively. The amplified products were separated on agarose gels (1.5%) and sequenced on an ABI 3730XL DNA analyzer. Mitochondrial gene sequences were subjected to multiple sequence alignment using ClustalW (http://www.clustal.org) with MEGA v. 5.1.

### Lectin histochemistry

Three lectin screening kits (I-III) were purchased from Vector Laboratories (Burlingame, CA, USA). Lectins were classified into five groups according to the binding specificity and inhibitory sugars, including N-acetylglucosamine, mannose, galactose/N-acetylgalactosamine, complex type N-glycan groups (PHA-E and PHA-L), and fucose (Table 1) as discussed previously [5, 7]. The following sugars were obtained from Sigma-Aldrich and used for competitive inhibition (see Table 1): α-methyl mannoside/α-methyl glucoside (Cat#, M6882, Sigma-Aldrich), β-D-galactose (Cat#, G6637, Sigma-Aldrich), lactose (Galβ1, 4Glc) (Cat#, L0100000, Sigma-Aldrich), melibiose (Galα1, 6Glc) (Cat#, M5500, Sigma-Aldrich), N-acetyl-D-galactosamine (α-D-GalNAc) (Cat#, A2795, Sigma-Aldrich), and N-acetyl-D-glucosamine (β-D-GlcNAc) (Cat#, A8625, Sigma-Aldrich). Chitin hydrolysate (Cat#, SP-0090, Vector) was purchased from Vector Laboratories.

**Table 1.** Binding specificities of lectins used in this study.

Lectin abbreviations	Source	Concentration (μg/mL)	Sugar specificity	Inhibitor or eluting sugar*
N-acetylglucosamine				
s-WGA	Succinylated-Wheat germ agglutinin	1.0 × 10 ^−2^	GlcNAc	0.2 M GlcNAc
WGA	Wheat germ agglutinin	1.0 × 10 ^−2^	GlcNAc NeuAc, SA	0.2 M GlcNAc
BSL-II	*Bandeiraea simplicifolia*	4.0 × 10^−3^	α or β GlcNAc	0.2 M GlcNAc
DSL	*Datura stramonium*	4.0 × 10^−3^	(GlcNAc)_2–4_	0.5 M chitin hydrolysate
LEL	*Lycopersicon esculentum*	2.0 × 10 ^−2^	(GlcNAc)_2–4_	0.5 M chitin hydrolysate
STL	*Solanum tuberosum*	1.0 × 10 ^−2^	(GlcNAc)_2–4_	0.5 M chitin hydrolysate
Mannose				
ConA	*Canavalia ensiformis*	3.3 × 10^−3^	αMan, αGlc, 4GlcNAc	0.2 M MeαMan/0.2 M MeαGlc
LCA	*Lens culinaris*	4.0 × 10^−3^	αMan, αGlc 4(Fucα1,6)GlcNAc	0.2 M MeαMan /0.2 M MeαGlc
PSA	*Pisum sativum*	4.0 × 10^−3^	αMan, αGlc 4(Fucα1,6)GlcNAc	0.2 M MeαMan/0.2 M MeαGlc
Galactose/N-acetylgalactosamine				
RCA_120_	*Ricinus communis*	2.0 × 10^−3^	Gal	0.2 M lactose
BSL-I	*Bandeiraea simplicifolia*	4.0 × 10^−3^	αGal, αGalNAc	0.2 M GalNAc
VVA	*Vicia villosa*	4.0 × 10^−3^	GalNAc	0.2 M GalNAc
DBA	*Dolichos biflorus*	1.0 × 10 ^−2^	αGalNAc	0.2 M GalNAc
SBA	*Glycine max*	1.0 × 10 ^−2^	α > βGalNAc	0.2 M GalNAc
SJA	*Sophora japonica*	2.0 × 10 ^−2^	βGalNAc	0.2 M GalNAc
Jacalin	*Artocarpus integrifolia*	5.0 × 10^−4^	Galβ3GalNAc	0.2 M melibiose
PNA	*Arachis hypogaea*	4.0 × 10^−3^	Galβ3GalNAc	0.2 M βGal
ECL	*Erythrina cristagalli*	2.0 × 10^−2^	Galβ4GlcNAc	0.2 M lactose
Complex type N-glycans (complex oligosaccharides)				
PHA-E	*Phaseolus vulgaris*	5.0 × 10^−3^	Galβ4GlcNAcβ 2 Man α6 (GlcNAcβ4)(GlcNAcβ4Manα 3) Manβ4	0.1 M acetic acid
PHA-L	*Phaseolus vulgaris*	2.5 × 10^−3^	Galβ4GlcNAcβ6 (GluNAcβ2Man α3) Man α3	0.1 M acetic acid
Fucose				
UEA-I	*Ulex europaeus*	2.0 × 10^−2^	αFuc	0.1 M L-fucose

Fuc, fucose; gal, galactose; GalNAc, N-acetylgalactosamine; glc, glucose; GlcNAc, N-acetylglucosamine; man, mannose. MeαMan, α-Methylmannoside; MeαGlc, α-Methylglucoside; NeuAc, N-acetylneuraminic acid; SA, sialic acid.

* The acronyms and lectin sepcificities including sources, preferred sugar specificities and inhibitor were obtained from the data sheet (Vector laboratory) and a previous paper [7].

Briefly, as in our previous study [12], the paraffin-embedded muscles were cut into 5 μm thick sections using a microtome. The sections were mounted on glass microscope slides, the paraffin was removed, and the sections were rehydrated. Endogenous peroxidase activity was blocked using 0.3% hydrogen peroxide in methanol for 30 minutes. After three washes with phosphate-buffered saline (PBS), the sections were incubated with 1% bovine serum albumin to block nonspecific binding. The sections were rinsed with PBS and incubated with the avidin-biotin-complex (ABC) method using 21 biotinylated lectins (Table 1) from the lectin screening kits I–III (Vector Laboratories) at 4 °C overnight. Horseradish peroxidase (HRP) was developed using a diaminobenzidine substrate kit (DAB Kit; Vector Laboratories). The sections were counterstained with hematoxylin before mounting. Negative controls for the lectin histochemistry included (1) omission of primary reagent (biotinylated lectins) and (2) preincubation of the lectins with the appropriate inhibitors (0.2 M–0.5 M in Tris buffer) for 1 hour at room temperature, as shown in a previous paper [7]. The intensities of the lectin-binding patterns on the slides were arbitrarily scored blind by three researchers as follows: − negative, ± occasionally weakly positive, + some, but not all, positive, ++ moderately positive, and +++ very strongly positive.

## Results and discussion

### Histological examination and PCR genotyping

**Figure 1.**
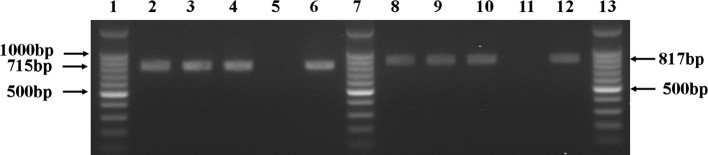
PCR amplification of the mitochondrial gene fragments (715 and 817 bp) from *Kudoa septempunctata* (in triplicate). Lanes: 1; SiZer^TM^-100 bp DNA Marker (iNtRON, Korea), 2–4; *cox 1* gene, 5; negative control, 6; positive control, 7; Molecular size marker, 8–10, *rnl* gene, 11; negative control (no DNA), 12; positive control, 13; M:SiZer^TM^-100 bp DNA Marker.

Muscles of olive flounder infected with *Kudoa* spp. showed sarcoplasmic infection with formation of pseudocysts. The infected muscle fibers were hypertrophied as shown in our previous report [2]. PCR analysis of the two mitochondrial genes *cox 1* and *rnl* of the *K. septempunctata* resulted in amplification of 751 bp (Fig. 1, lane 2–4) and 817 bp fragments (Fig. 1, lane 8–10), respectively, matching with the results of histopathology. The obtained gene sequences were subjected to multiple sequence alignment using ClustalW (http://www.clustal.org). Aligned fragments showed high sequence similarity (100%) with the type strains LC014799 and AB915832, which revealed that the isolated *K. septempunctata* belonged to the ST3 genotype [2].

### Lectin histochemistry

In the hypertrophied muscle fibers, pseudocysts contained spores at two different stages, i.e., sporoblasts and mature spores. In the present study, we did not discriminate between mature spores and immature sporoblasts because they are morphologically indistinguishable under light microscopy. In the *K. septempunctata*-infected tissues, transmission electron microscopic analysis revealed that both mature spores and immature sporoblasts were encased by a plasmodial membrane [4, 11].

The 21 lectins examined belonged to the following groups; N-acetylglucosamine (s-WGA, WGA, BSL-II, DSL, LEL, and STL), mannose (Con A, LCA, and PSA), galactose/N-acetylgalactosamine (RCA_120_, BSL-I, VVA, DBA, SBA, SJA, Jacalin, PNA, and ECL), oligosaccharides (PHA-E and PHA-L), and fucose (UEA-I) (Table 1).

In the present study, we used a more diverse range of lectins (Table 1). Lectin reactivity was scored on spores and plasmodial membranes, but specific discrimination of polar capsules and valves of spores was not performed because of the limitations of identification by light microscopy. In addition, we did not discriminate between mature type spores and immature sporoblasts because both types were present in the same pseudocyst.

**Figure 2.**
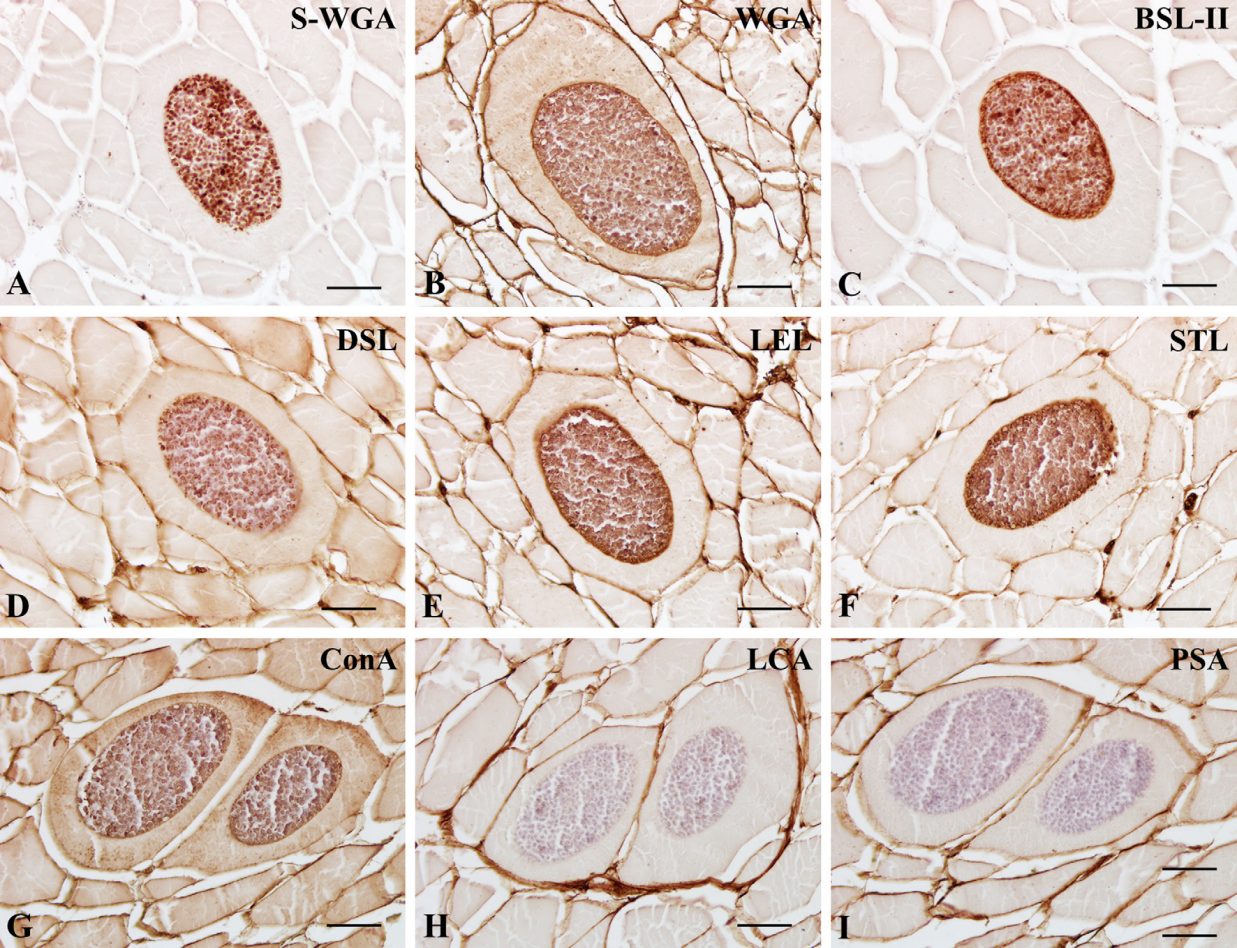
Lectin histochemical findings of N-acetylglucosamine (S-WGA, WGA, BSL-II, DSL, LEL, STL) and mannose groups (Con A, LCA, PSA) in *Kudoa septempunctata*-infected muscles. A, S-WGA; B, WGA; C, BSL-II; D, DSL; E, LEL; F, STL; G, Con A; H, LCA; I, PSA. Scale bars in (A)–(I) represent 20 μm.

**Figure 3.**
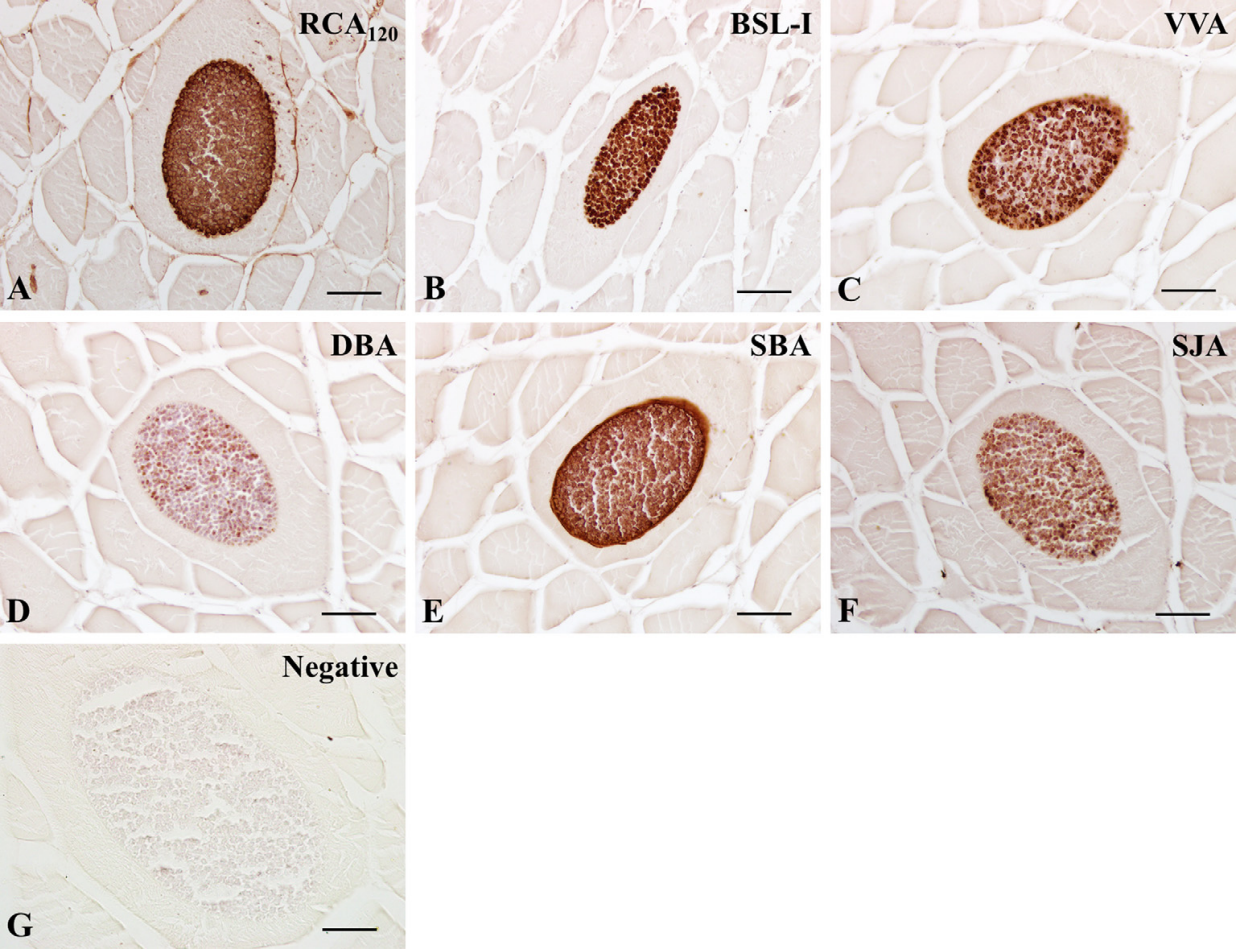
Lectin histochemical findings of galactose/N-acetylgalactosamine in *Kudoa septempunctata*-infected muscles. A, RCA120; B, BSL-I; C, VVA; D, DBA; E, SBA; F, SJA; G, Negative control (DBA preabsorbed with 0.2 M GalNAc). Scale bars in (A)–(G) represent 20 μm.

**Figure 4.**
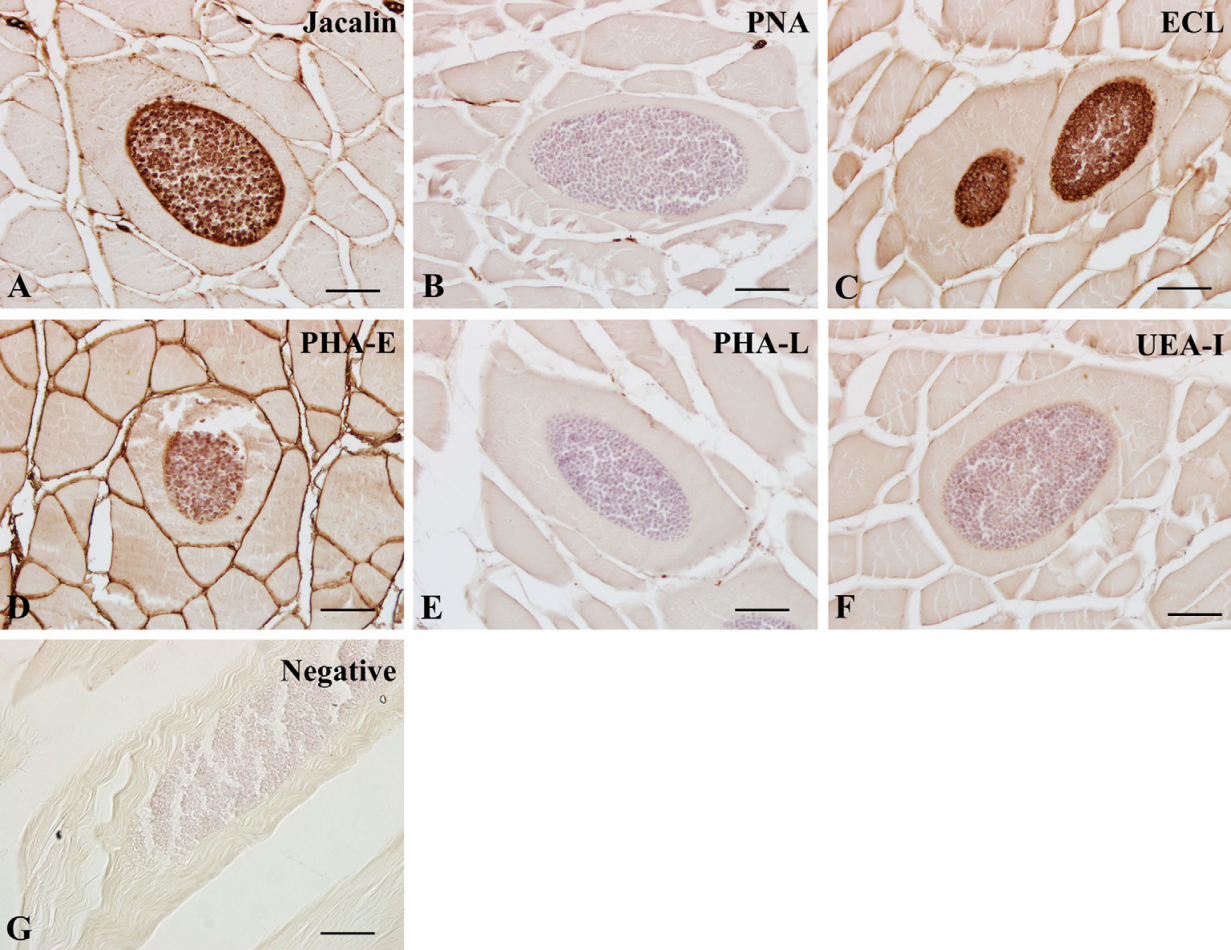
Lectin histochemical findings of galactose/N-acetylgalactosamine (Jacalin, PNA, ECL), oligosaccharide (PHA-E, PHA-L), and fucose groups (UEA-I) in *Kudoa septempunctata*-infected muscles. A, Jacalin; B, PNA; C, ECL; D, PHA-E; E, PHA-L; F, UEA-I; G, Negative control (UEA-I preabsorbed with 0.1 M L-fucose). Scale bars in (A)–(G) represent 20 μm.

In *K. septempunctata*-infected muscles, the majority of lectins, except LCA, PSA, PNA, and PHA-L, were positive in spores, while lectin reactivity on the plasmodial membrane encasing clusters of spores mostly matched those of spores with varying intensities (Figs. 2–4). These findings suggested that spores contain a variety of carbohydrate groups on their surfaces, including N-acetylglucosamine (Fig. 2), mannose (Fig. 2), galactose/N-acetylgalactosamine (Figs. 3 and 4), and fucose groups (Fig. 4), with different intensities of each lectin (Table 2). Due to the specificity of RCA_120_, DSL-I, DBA, and SJA on spores, these lectins may be candidates for *Kudoa* markers, at least for *K. septempunctata*.

**Table 2.** Lectin binding patterns of spores in the muscles of olive flounder infected by *Kudoa septempunctata*.

Lectin	Olive flounder			*Kudoa septempunctata*	
	Epidermis	Endomysium	Sarcoplasma (non-infected/infected)	Spore	Plasmodial membrane
N-acetylglucosamine					
s-WGA	+	−	−/−	+++	±
WGA	+++	++	−/+	++	++
BSL−II	−	−	−/−	+++	++
DSL	+++	+	+/+	++	+
LEL	+++	++	−/−	++	++
STL	+++	+	−/−	++	+
Mannose					
ConA	+++	+	+/++	+	+
LCA	++	++	−/−	−	−
PSA	+++	++	−/−	−	−
Galactose/N-acetylgalactosamine					
RCA_120_	++	−	−/−	+++	−
BSL-I	+	−	−/−	+++	−
VVA	+++	−	−/−	+++	+
DBA	−	−	−/−	+	−
SBA	+	−	−/−	+++	++
SJA	−	−	−/−	++	−
Jacalin	+++	+	+/+	+++	++
PNA	+++	−	−/−	−	−
ECL	−	+	+/+	+++	+
PHA-E	+++	++	+/+	+++	+
PHA-L	+++	−	−/−	−	−
Fucose					
UEA-I	+++	−	−/−	± (weak)	±

*Note*. −; negative, ±; occasionally weak positive, +; some, but not all, positive, ++; moderately positive, +++; very strong positive.

Even though Con A, LCA, and PSA belong to the same mannose group, we found that Con A (Fig. 2G), but not LCA (Fig. 2H) and PSA (Fig. 2I), labeled on spores (Table 1). We postulate that a minor difference in preferred oligosaccharide 4GlcNAc of Con A would be one of the factors because 4(Fucα 1,6)GlcNAc is a preferred oligosaccharide in LCA and PSA (see Table 1 in this report and Table 1 [7]). With regard to the reactivity of oligosaccharides, PHA-E, but not PHA-L, labeled spores, the plasmodial membrane, and the sarcoplasm, suggesting that the same oligosaccharide group shows distinct patterns in *K. septempunctata*-infected tissues. We postulate that some lectins with the same sugar specificities label different carbohydrate residues.

With regard to the differences in lectin reactivity on spores and the plasmodial membrane in this study, we postulate that carbohydrates on the plasmodial membrane originated largely from spores, while some were modified in the plasmodial membrane because lectin reactivity of DBA and SJA was not found in the plasmodial membrane despite their presence on spores. We do not exclude the possibility that some carbohydrate residues are used for penetration of the parasite into the host because carbohydrate residues are known to act as linkers between host cells and parasites [13, 14].

It has been reported that the addition of glucose to the culture medium plays an important role in the release of *K. septempunctata* sporoplasm [16], suggesting that glucose mediates disruption of the sporoplasm. However, it is unclear which factors are involved in human diarrhea, because spores do not induce diarrhea in adult mice [2]. We cannot rule out the possibility that sporoplasm of *Kudoa* spp. may disturb the intestinal microorganisms in some human subjects.

For the sarcoplasm, we compared lectin reactivity of infected muscle fibers with non-infected fibers in the same tissue sections. The majority of the sarcoplasm in non-infected fibers was negative for lectins, except DSL, Con A, Jacalin, ECL, and PHA-E. We postulate that hypertrophy in spore-infected muscle fibers is not directly related to carbohydrate residues.

Some lectins, i.e., WGA, DSL, LEL, STL, Con A, LCA, PSA, Jacalin, ECL, and PHA-E, were found to be positive on the endomysium, while others were not positive in this study. We postulate that interstitial connective tissues are not changed after *K. septempunctata* infection.

The epidermis also showed reactivity for the majority of lectins, except BSL-II, DBA, and SJA (Table 2), suggesting that a variety of carbohydrate residues cover flat fish skin. Even in the absence of BSL-II, DBA, and SJA reactivity in the epidermis, they were positive in spores. Conversely, LCA, PAS, and PNA were negative on spores but positive on the epidermis. In a limited examination of lectin binding in flat fish [6], it was found that each lectin labels some epithelial cells and mucus cells in flat fish with varying intensities, suggesting that carbohydrate residues are present, but no examination of the epidermis was performed. In the present study, we found that a variety of lectin labelings were localized on the epidermis, suggesting that all types of carbohydrates (N-acetylglucosamine, mannose, galactose/N-acetylgalactosamine, complex type N-glycans oligosaccharides (PHA-E and PHA-L), and fucose groups) exist on the skin of flat fish. We postulate that a variety of carbohydrate residues are constitutively expressed on the epidermis, possibly contributing to protection against environmental stimuli in this species.

Taken together, these observations suggest that BSL-II, DBA, and SJA are specific markers for spores of *K. septempunctata* ST3, and that certain carbohydrate residues in the spores would be important molecules for survival of this parasite.

## Conflict of interest

The authors declare no conflicts of interest.
